# Antibody-Dependent Enhancement of SARS-CoV-2 Infection Is Mediated by the IgG Receptors FcγRIIA and FcγRIIIA but Does Not Contribute to Aberrant Cytokine Production by Macrophages

**DOI:** 10.1128/mBio.01987-21

**Published:** 2021-09-28

**Authors:** Tadashi Maemura, Makoto Kuroda, Tammy Armbrust, Seiya Yamayoshi, Peter J. Halfmann, Yoshihiro Kawaoka

**Affiliations:** a Department of Pathobiological Sciences, School of Veterinary Medicine, University of Wisconsin-Madison, Madison, Wisconsin, USA; b Division of Virology, The Institute of Medical Science, The University of Tokyo, Tokyo, Japan; c The Research Center for Global Viral Diseases, National Center for Global Health and Medicine Research Institute, Tokyo, Japan; d Department of Special Pathogens, International Research Center for Infectious Diseases, The Institute of Medical Science, The University of Tokyo, Tokyo, Japan; St. Jude Children's Research Hospital

**Keywords:** ADE, antibody-dependent enhancement, COVID-19, FcγRIIA, FcγRIIIA, SARS-CoV-2, macrophages

## Abstract

The coronavirus disease 2019 (COVID-19) pandemic has raised concerns about the detrimental effects of antibodies. Antibody-dependent enhancement (ADE) of infection is one of the biggest concerns in terms of not only the antibody reaction to severe acute respiratory syndrome coronavirus 2 (SARS-CoV-2) upon reinfection with the virus but also the reaction to COVID-19 vaccines. In this study, we evaluated ADE of infection by using COVID-19 convalescent-phase plasma and BHK cells expressing human Fcγ receptors (FcγRs). We found that FcγRIIA and FcγRIIIA mediated modest ADE of infection against SARS-CoV-2. Although ADE of infection was observed in monocyte-derived macrophages infected with SARS-CoV-2, including its variants, proinflammatory cytokine/chemokine expression was not upregulated in macrophages. SARS-CoV-2 infection thus produces antibodies that elicit ADE of infection, but these antibodies do not contribute to excess cytokine production by macrophages.

## INTRODUCTION

Severe acute respiratory syndrome coronavirus 2 (SARS-CoV-2), the causative agent of coronavirus disease 2019 (COVID-19), has spread rapidly around the world and caused a devastating pandemic (1); as of May 2021, there have been more than 166,220,000 cases and 3,445,000 deaths worldwide (2). The SARS-CoV-2 and its variants continue to ravage human health and the global economy.

During a pandemic, a global vaccination campaign is essential to mitigate the risk of infection and spread (3). To date, several vaccines have been developed and approved (4). However, one of the biggest safety concerns with vaccines is a phenomenon known as antibody-dependent enhancement (ADE) of virus infection (5). ADE of infection should also be a consideration when patients are being treated with convalescent-phase plasma or monoclonal antibodies (5). Moreover, with the emergence of SARS-CoV-2 variants, the risk for reinfection also raises the possibility of ADE of infection.

ADE is an alternative mechanism of virus infection of cells (5–7). An immune complex of virus and antibodies (mostly nonneutralizing antibodies or cross-reactive antibodies) can bind to receptor molecules, called Fcγ receptors (FcγRs), on immune cells and be internalized, which leads to enhancement of virus entry (5, 7). Because macrophages/monocytes express FcγRs (FcγRIA, FcγRIIA, and FcγRIIIA) on their surfaces (7–9), macrophages are considered the major inducers of ADE of infection. Moreover, hyperinflammation is often caused by immune cells, including macrophages, upon ADE of various viral infections (10).

ADE of infection occurs with a variety of viruses, including dengue virus, respiratory syncytial virus, and influenza virus, as well as the coronaviruses SARS-CoV-1 and Middle East respiratory syndrome coronavirus (MERS-CoV) (5, 6). Several studies have been performed to investigate whether SARS-CoV-2 infection induces ADE of infection (11, 12), and ADE of SARS-CoV-2 infection was observed in a study of convalescent-phase-plasma therapy (12). While FcγRIIA was reported to mediate ADE of SARS-CoV-2 infection in that study, the precise mechanism was not fully elucidated. In addition, it remains unclear whether FcγRIA and FcγRIIIA are involved in ADE of SARS-CoV-2 infection, although they have been reported to mediate ADE of infection with porcine reproductive virus and respiratory syndrome virus (13) and with dengue virus (14) and Japanese encephalitis virus (15), respectively. Moreover, it is not known whether ADE of SARS-CoV-2 infection elicits abnormal cytokine productions in macrophages or whether ADE of infection is induced with SARS-CoV-2 variants.

To address these unknowns, here, we investigated the mechanism of ADE of SARS-CoV-2 infection by using convalescent-phase plasma from COVID-19 patients and found that ADE of infection is mainly mediated by two types of FcγRs: FcγRIIA and FcγRIIIA.

## RESULTS AND DISCUSSION

### SARS-CoV-2 infection induces antibodies that elicit ADE of infection.

We first examined whether FcγRs *per se* mediate SARS-CoV-2 entry. We generated BHK cells stably expressing human FcγRs (FcγRIA, FcγRIIA, or FcγRIIIA) or human angiotensin-converting enzyme 2 (hACE2) (the entry receptor for SARS-CoV-2). Because wild-type BHK cells lack human ACE2 expression and are not susceptible to SARS-CoV-2 (16), these cells could be used to test whether these transfected proteins mediate ADE of SARS-CoV-2 infection. BHK cells were infected with a firefly luciferase-expressing vesicular stomatitis virus (VSV) lacking the VSV-G gene and pseudotyped with SARS-CoV-2 spike (VSV-SARS2-S). Cells were lysed, and luciferase activity was evaluated at 24 h postinfection (hpi). As expected, although BHK-hACE2 cells were susceptible to VSV-SARS2-S, the BHK-FcγRIA, BHK-FcγRIIA, and BHK-FcγRIIIA cells were not susceptible due to the lack of hACE2 (see Fig. S1A in the supplemental material) (16). Next, we tested whether plasma from COVID-19 patients mediated ADE of SARS-CoV-2 infection. We used 15 convalescent-phase plasma samples randomly selected from 110 plasma samples (listed in Fig. S2A and B) and one plasma sample from an uninfected individual. BHK-FcγRIA, BHK-FcγRIIA, and BHK-FcγRIIIA cells were infected with VSV-SARS2-S that was incubated with the serially diluted plasma samples, and the luciferase signal was assessed at 24 hpi. We did not detect any luciferase signals in any samples, suggesting that BHK cells expressing FcγRs *per se* do not mediate ADE of SARS-CoV-2 infection (Fig. S1B to D).

10.1128/mBio.01987-21.1 FIG S1 ADE of SARS-CoV-2 infection is not mediated by FcγRs *per se.* (A) The indicated cells were infected with or without VSV-SARS2-S, and luciferase activities in cell lysates were determined at 24 hpi. The experiment was performed with duplicate samples; means and SD are shown. (B) Serially diluted convalescent-phase plasma samples from 15 individuals or control plasma incubated with VSV-SARS2-S were used to infect the indicated cells, and the luciferase activity in the cell lysates was determined at 24 hpi. Download FIG S1, TIF file, 2.2 MB. Copyright © 2021 Maemura et al. 2021 Maemura et al. https://creativecommons.org/licenses/by/4.0/ This content is distributed under the terms of the Creative Commons Attribution 4.0 International license.

10.1128/mBio.01987-21.2 FIG S2 Screening for ADE-inducing antibodies in COVID-19 convalescent-phase plasma. (A) Serially diluted convalescent-phase plasma samples from 110 individuals and two control plasma samples that were incubated with VSV-SARS2-S were used to infect the indicated cells that had been transfected with an hACE2 expression vector; the luciferase activity in the cell lysates was determined at 24 hpi. The 110 plasma samples were randomly divided into six groups. Gray lines indicate plasma from patients; blue lines indicate control plasma. (B and C) Convalescent-phase plasma samples from the indicated individuals and control plasma diluted 1:1,600 and incubated with VSV-SARS2-S were used to infect (B) BHK-FcγRIIA cells or (C) BHK-FcγRIIIA/FCER1G cells transfected with an hACE2 expression vector; luciferase activity in cell lysates was determined at 24 hpi. Statistical analysis was performed by using a one-way ANOVA followed by Dunnett’s test. Plasma samples that showed a significant increase in luciferase signal compared to the control are indicated with asterisks (**, *P < *0.01; *, *P < *0.05). The experiments were performed in duplicate; means and SD are shown. Download FIG S2, PDF file, 1.4 MB. Copyright © 2021 Maemura et al. 2021 Maemura et al. https://creativecommons.org/licenses/by/4.0/ This content is distributed under the terms of the Creative Commons Attribution 4.0 International license.

**FIG 1:**
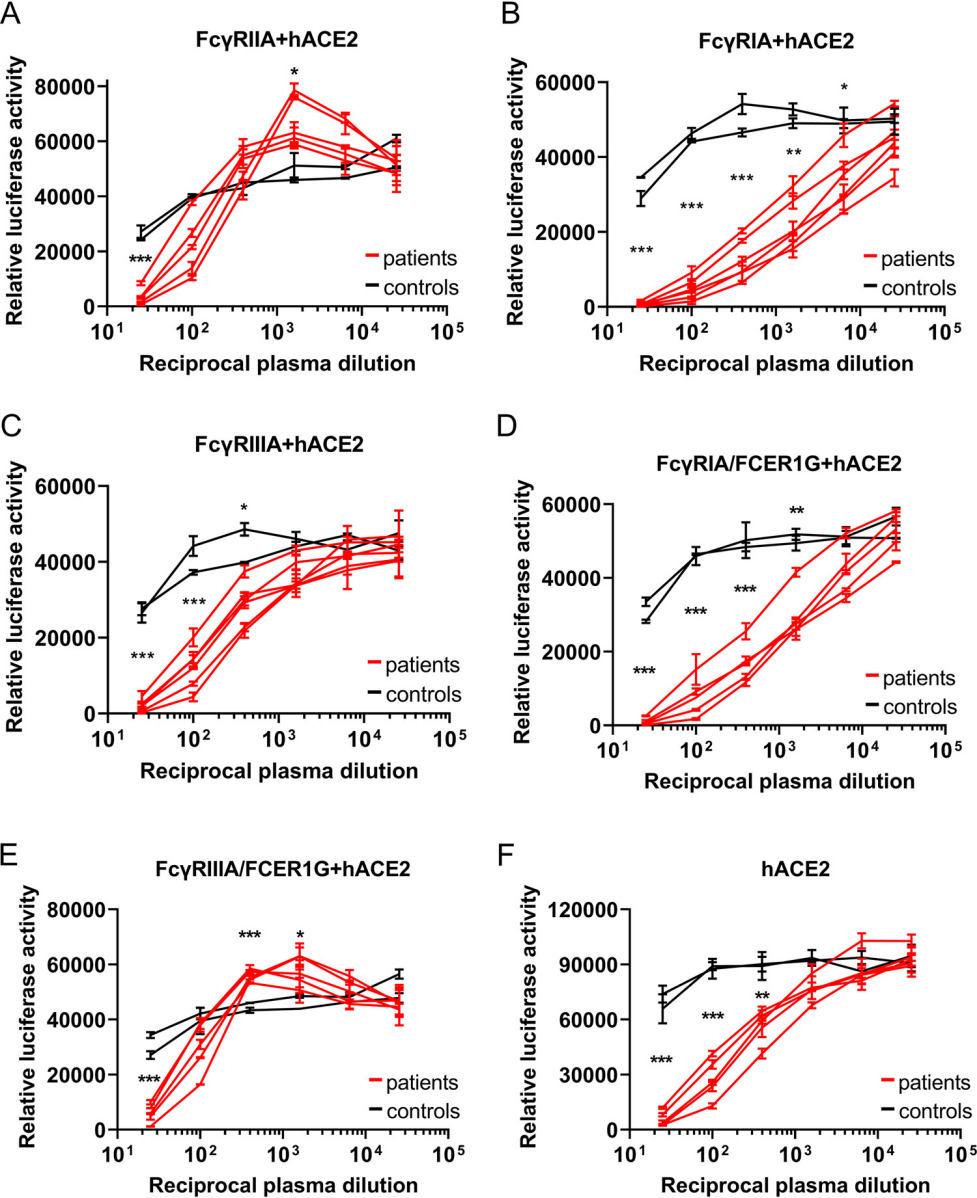
ADE of SARS-CoV-2 infection is mainly mediated by FcγRIIA and FcγRIIIA. (A to E) Serially diluted convalescent-phase plasma from five individuals and two control plasma samples incubated with VSV-SARS2-S were used to infect the indicated cells that had been transfected with an hACE2 expression vector; the luciferase activity in the cell lysates was determined at 24 hpi. The experiment was performed with duplicate samples; means and standard deviations (SD) are shown. (F) Serially diluted convalescent-phase plasma from two individuals and two control plasma samples incubated with VSV-SARS2-S were used to infect the indicated cells, and the luciferase activity in the cell lysates was determined at 24 hpi. The experiments were performed in duplicate; means and SD are shown. Statistical analysis was performed using an unpaired *t* test. ***, *P < *0.001; **, *P < *0.01; *, *P < *0.05.

Next, we tested whether ADE of infection was elicited in the presence of hACE2. We transfected BHK-FcγRIIA cells with a hACE2 expression vector, infected them with VSV-SARS2-S that had been incubated with the serially diluted plasma, and evaluated the luciferase signals. We screened 110 plasma samples from COVID-19 patients. These samples were randomly divided into six groups (see Fig. S2A for complete results of the screen). In Fig. 1, we show the results from the five convalescent-phase plasma samples that showed the highest luciferase signals at a 1:1,600 dilution (compared to the control in Fig. S2A) and two control plasma samples as representative data. We found that the luciferase levels were significantly lower for the plasma from COVID-19 patients (Fig. 1A, red lines) under 1:25-diluted conditions compared to control plasma (black lines), which indicates neutralization of VSV-SARS2-S. In contrast, the luciferase levels were significantly higher under the 1:1,600-dilution conditions with plasma from COVID-19 patients, indicating that the VSV-SARS2-S infection was enhanced by the convalescent-phase plasma via FcγRIIA in the presence of ACE2 (Fig. 1A). We then evaluated ADE of infection in BHK-FcγRIA and BHK-FcγRIIIA cells transfected with hACE2 by using the same five plasma samples that induced ADE of infection in BHK-FcγRIIA cells, and found that ADE was not elicited via FcγRIA or FcγRIIIA even in the presence of ACE2 (Fig. 1B and C).

A previous study reported that an association with the FcRγ subunit (Fc fragment of IgE receptor Ig [FCER1G]) is required for activation and function of FcγRIA and FcγRIIIA at the cell surface (8). We therefore engineered BHK-FcγRIA and BHK-FcγRIIIA cells to stably express FCER1G. Next, we evaluated ADE of infection in the BHK-FcγRIA/FCER1G and BHK-FcγRIIIA/FCER1G cells transfected with an hACE2 expression vector. Although we did not detect ADE of infection in BHK-FcγRIA/FCER1G cells (Fig. 1D), we did observe a significant increase in the luciferase signals in BHK-FcγRIIIA/FCER1G cells with the patient plasma at 1:400 to 1:1,600 dilutions, indicating that the infection by VSV-SARS2-S was enhanced by convalescent-phase plasma not only via FcγRIIA but also via FcγRIIIA (Fig. 1E). Moreover, we did not detect ADE of infection in BHK cells solely expressing hACE2 (Fig. 1F). Taken together, our data show that SARS-CoV-2 infection induces antibodies that elicit ADE of infection in humans. ADE of SARS-CoV-2 infection was observed via FcγRIIA and FcγRIIIA in the presence of hACE2.

Next, we expanded our evaluation of ADE of infection to 90 plasma samples with BHK-FcγRIIIA/FCER1G cells in the presence of hACE. Because we observed the highest luciferase signals with 1:1,600-diluted plasma (Fig. S2A; Fig. 1A and E), we screened the ADE-inducible plasma samples under this experimental condition. We found that 19 (17.3%) and 15 (16.7%) plasma samples significantly increased the luciferase signals compared to the control plasma samples in BHK-FcγRIIA and BHK-FcγRIIIA/FCER1G cells, respectively (Fig. S2B and C). Of the plasma tested, 6 (6.7%) induced ADE of infection via both FcγRIIA and FcγRIIIA/FCER1G.

### Antibodies that induce ADE of infection are present for at least 6 months after infection.

**FIG 2:**
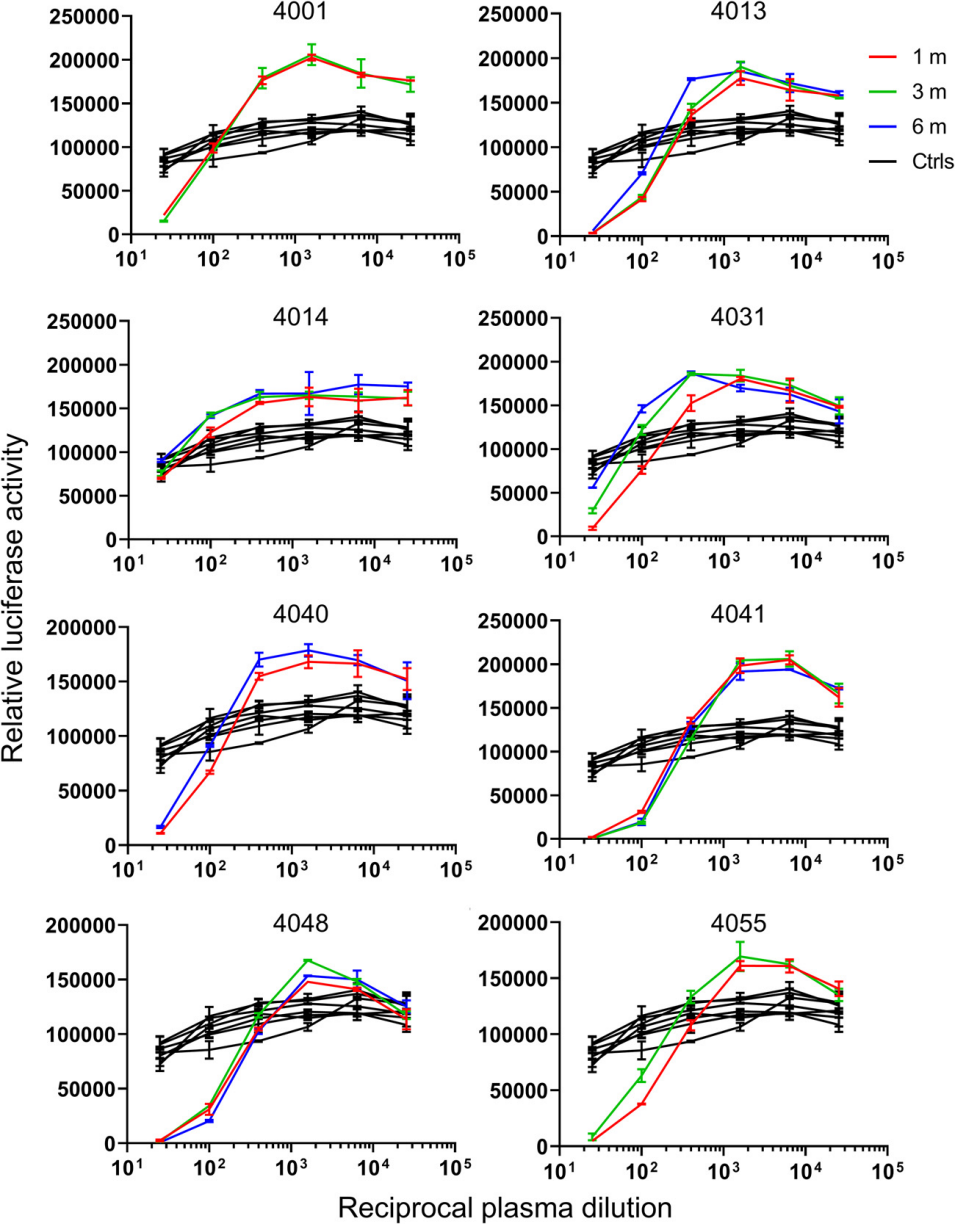
Antibodies that induce ADE of infection exist for at least 6 months after infection. Serially diluted convalescent-phase plasma samples (obtained at 1, 3, and 6 months after diagnosis) from the indicated individuals and seven control plasma samples that had been incubated with VSV-SARS2-S were used to infect BHK-FcγRIIA cells that had been transfected with the hACE2 expression vector; luciferase activity in the cell lysates was determined at 24 hpi. The experiments were performed in duplicate; means and SD are shown.

We obtained convalescent-phase plasma from COVID-19 patients at 1, 3, and 6 months after diagnosis. We could therefore investigate the duration of antibodies that induce ADE of infection in COVID-19 patients. We selected eight plasma samples (4001, 4013, 4014, 4031, 4040, 4041, 4048, and 4055) that were positive for ADE of infection via FcγRIIA (Fig. S2A and B). BHK-FcγRIIA cells transfected with hACE2 expression plasmids were infected with VSV-SARS2-S that had been incubated with serially diluted plasma, and luciferase levels were evaluated at 24 hpi. Plasma collected at 3 or 6 months after diagnosis increased the luciferase signals to levels identical to those seen with plasma collected at 1 month after diagnosis (Fig. 2), indicating that ADE-inducing antibodies may exist for at least 6 months after SARS-CoV-2 infection.

### SARS-CoV-2 infection is enhanced by convalescent-phase plasma in primary macrophages.

**FIG 3:**
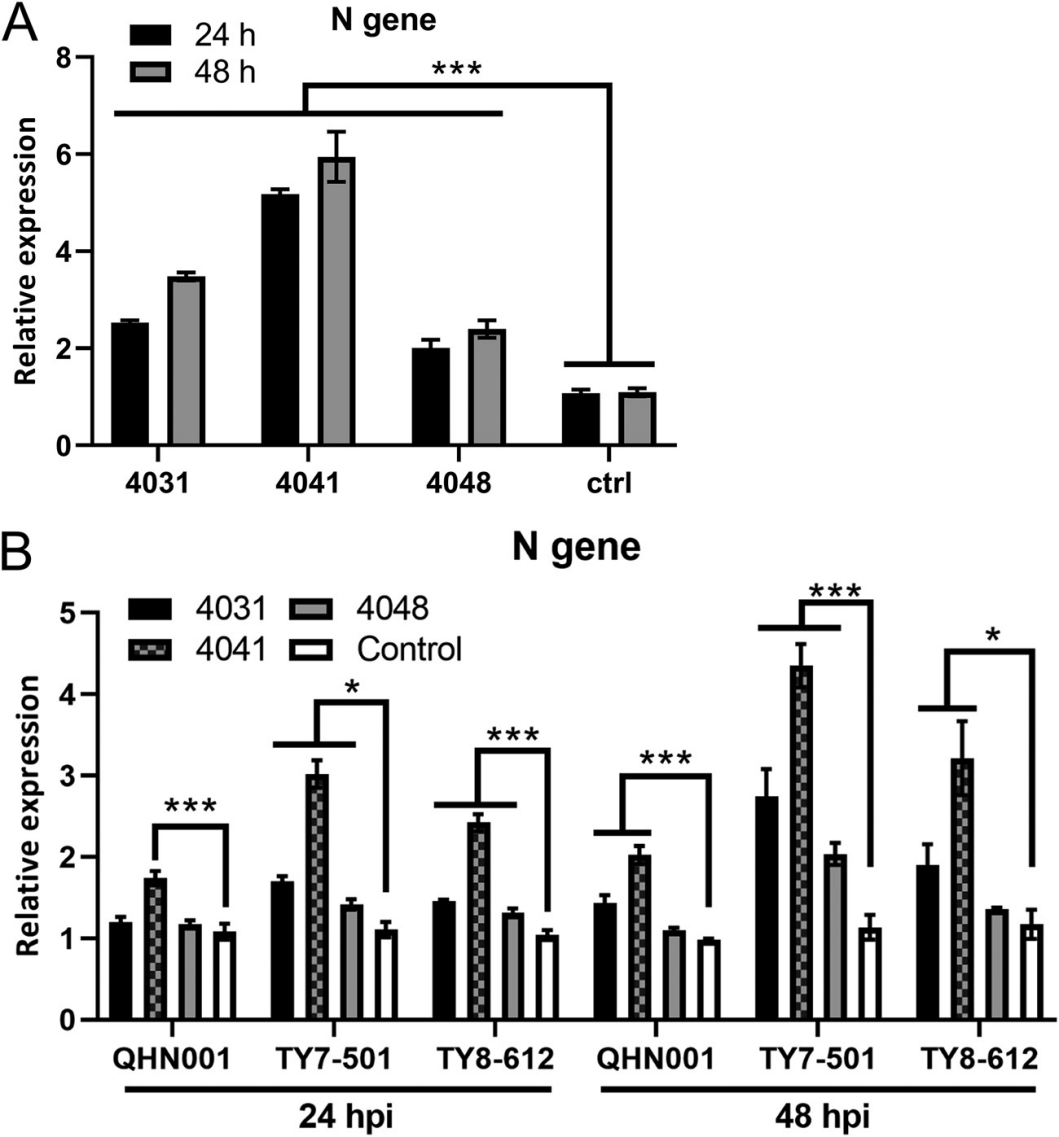
SARS-CoV-2 infection is enhanced by convalescent-phase plasma in primary macrophages. Three representative ADE-inducing plasma samples (4031, 4041, and 4048) and control plasma were used for these experiments. Monocyte-derived macrophages were infected at an MOI of 1 with (A) NCGM02 or (B) QHN001, TY7-501, or TY8-612 that had been incubated with the indicated plasma diluted to 1:1,600. Total RNA was isolated from cells at 24 and 48 hpi. The N gene was quantified by RT-qPCR. Results are presented relative to the control plasma-treated cell levels (2^−ΔΔ^*^CT^*). Statistical analysis was performed by using a one-way analysis of variance (ANOVA) followed by Dunnett’s test. ***, *P < *0.001; *, *P < *0.05. The experiments were performed in triplicate; means and SD are shown.

Macrophages endogenously express FcγRs (7–9). Therefore, to investigate whether ADE of infection is elicited in primary human macrophages, we infected monocyte-derived macrophages with authentic SARS-CoV-2 (NCGM02) that had been incubated with 1:1,600-diluted convalescent-phase plasma. We selected three convalescent-phase plasma samples that elicited ADE via FcγRIIA and FcγRIIIA/FCER1G (4031, 4041, and 4048) as representative plasma for this experiment. RNA was isolated from cells at 24 and 48 hpi, and reverse transcription-quantitative PCR (RT-qPCR) was performed to quantify the viral N genes. We found that N gene expression was significantly increased in macrophages incubated with convalescent-phase plasma at 24 and 48 hpi (Fig. 3A). The patients in our cohort were diagnosed with COVID-19 in April 2020, which indicates that they were infected with early SARS-CoV-2 strains. Accordingly, next we investigated whether these convalescent-phase plasmas induce ADE of infection against recent SARS-CoV-2 variants. We used three variants (VOC202012/01, or B.1.1.7 [QHN001]; VOC202101/02, or P.1 [TY7-501]; and VOC202012/02, or B.1.351 [TY8-612]) and repeated the experiment we had performed with the early strain NCGM02. We found that macrophages infected with variants incubated with convalescent-phase plasma showed high levels of N genes compared to those incubated with control plasma (Fig. 3B). These results indicate that convalescent-phase plasma collected from patients infected with early SARS-CoV-2 strains also elicits ADE of infection against SARS-CoV-2 variants, although the increase in the level of N gene expression induced by ADE of infection tended to be lower in the variants.

### Contribution of ADE of SARS-CoV-2 infection to cytokine expression in macrophages.

**FIG 4:**
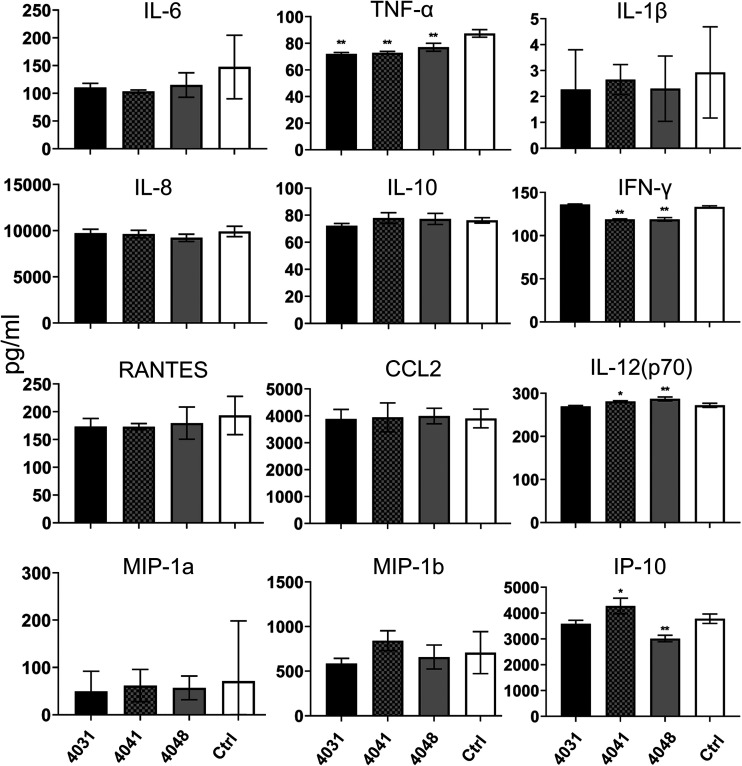
ADE of SARS-CoV-2 infection and cytokine expression in macrophages. Three representative ADE-inducing plasma samples (4031, 4041, and 4048) and control plasma were used for these experiments. Monocyte-derived macrophages were infected at an MOI of 1 with NCGM02 that had been incubated with the indicated plasma diluted to 1:1,600. Supernatant was collected at 24 hpi and analyzed for cytokine expression. Statistical analysis was performed by using a one-way ANOVA followed by Dunnett’s test. **, *P < *0.01; *, *P < *0.05. Data are means and SD.

COVID-19 induces the hyperinflammatory state in severe cases, which is also referred to as abnormal production of cytokines, such as interleukin 6 (IL-6), IL-8, IL-10, tumor necrosis factor alpha (TNF-α), and CCL2, in immune cells, including macrophages (17–20). Zheng et al. showed that gene expression of proinflammatory cytokines is upregulated in monocyte-derived macrophages after SARS-CoV-2 infection (21). Because we found that convalescent-phase plasma enhances SARS-CoV-2 infection, we evaluated whether ADE of SARS-CoV-2 infection contributes to inflammatory cytokine expression in macrophages. We infected monocyte-derived macrophages with NCGM02 SARS-CoV-2 that had been incubated with 1:1,600-diluted convalescent-phase plasma (4031, 4041, and 4048). The macrophages were infected with NCGM02 at a multiplicity of infection (MOI) of 1.0, and supernatant was collected at 24 hpi and analyzed for cytokine/chemokine profiles. We found that the expression levels of most inflammatory cytokines/chemokines were not altered by the ADE-inducing plasma relative to the controls, with the exception of a very few cytokines (Fig. 4). These results indicate that ADE-inducing antibodies may not contribute to aberrant cytokine production in macrophages.

In this study, we evaluated ADE-inducing antibodies in convalescent-phase plasma against SARS-CoV-2. We evaluated three major activating types of FcγRs (FcγRIA, FcγRIIA, and FcγRIIIA), which are expressed on monocytes/macrophages (7–9); FcγRIIIA is also expressed on natural killer cells. We used BHK cells as a model to evaluate enhancement of infection via FcγRs. Although these cells did not elicit ADE of infection (Fig. S1B to D), it was interesting to find that hACE2, as well as FcγRIIA and FcγRIIIA, was required to mediate ADE of SARS-CoV-2 infection in BHK cells (Fig. 1A and E); this finding suggest that FcγRIIA and FcγRIIIA may function as coreceptors upon ADE of infection. Of note, FcγR-mediated ADE of infection of SARS-CoV-2 was modest compared with that of dengue virus, which is known to induce robust ADE (22). ADE of infection of SARS-CoV-2 was also identified in primary macrophages (Fig. 3A), indicating that hACE2 is expressed on monocyte-derived macrophages, as well as FcγRs, which has been reported previously (12).

The emergence of several SARS-CoV-2 variants prompted us to assess the risk for reinfection with SARS-CoV-2, because these variants’ antigenicity has been reported to differ from that of early strains (23, 24). ADE of infection was identified for variants in this study (Fig. 3B). In our cohort samples, ADE of infection was observed only in plasma diluted more than 1:400, and strong neutralizing activity was found with lower dilutions (Fig. 2). These results indicate that neutralization may occur with plasma containing sufficient neutralizing antibodies but that ADE-inducing antibodies may function at lower concentrations than neutralizing antibodies. Given that recent studies have shown that neutralizing antibodies against SARS-CoV-2 S protein can exist for up to 8 months (25, 26), ADE-inducing antibodies may not elicit ADE of infection for several months. Our knowledge of antibody populations and duration in COVID-19 vaccine recipients remains limited. Recent studies have revealed a novel mechanism of ADE of SARS-CoV-2 infection that is not FcγR mediated (27, 28). These studies suggest that the antibodies produced in response to the vaccines that were developed based on early strains of SARS-CoV-2 could elicit ADE of infection for recent variants, including B.1.617.2 (delta) (27, 28). Additional studies are needed to evaluate how long ADE-inducing and neutralizing antibodies exist in vaccine recipients.

A recent study suggested that there is a correlation between ADE-inducing antibodies and COVID-19 disease severity (11). It has also been reported that hypercytokinemia—that is, the abnormal release of inflammatory cytokines from macrophages—occurs in COVID-19 patients (17–20). To investigate whether ADE of infection contributes to hypercytokinemia, we examined inflammatory cytokine release from macrophages incubated with ADE-inducing plasma. However, we found that inflammatory cytokine levels were not increased in macrophages incubated with ADE-inducing plasma. These results suggest that ADE-inducing antibodies do not function as inducers of inflammation but may function as antivirals, trapping the viruses in the macrophages; of note, no SARS-CoV-2 replication was observed in macrophages in our experiments (data not shown), which is consistent with previous studies (21, 29).

In conclusion, our study revealed that SARS-CoV-2 infection induces antibodies that elicit ADE of infection and ADE-inducing antibodies exist for at least 6 months after SARS-CoV-2 infection in humans. Although this ADE of infection was mainly mediated by FcγRIIA and FcγRIIIA, detrimental contributions by macrophages were not observed. Longitudinal studies are needed to evaluate the effect of ADE-inducing antibodies in SARS-CoV-2 infection.

## MATERIALS AND METHODS

### Ethics statement.

Plasma samples were obtained from deidentified participants under a protocol reviewed by the Human Subjects Institutional Review Boards at the University of Wisconsin—Madison.

### Isolation of convalescent-phase plasma from patients.

Blood samples were collected in EDTA blood collection tubes from 110 patients who had recovered from SARS-CoV-2 infection. Plasma samples were isolated by using Ficoll reagent according to the manufacturer’s instructions and stored at −80°C until use. Plasma samples from healthy donors collected before 2018 were purchased from Zenbio.

### Cells.

BHK (baby hamster kidney) cells stably expressing human FcγRIA (BHK-FcγRIA), FcγRIIA (BHK-FcγRIIA), FcγRIIIA (BHK-FcγRIIIA), FcγRIA and FCER1G (BHK-FcγRIA/FCER1G), FcγRIIIA and FCER1G (BHK-FcγRIIIA/FCER1G), and human ACE2 (BHK-hACE2) were generated as follows: a cDNA fragment encoding FcγRIA, FcγRIIA, FcγRIIIA, hACE2, or FCER1G was cloned into the murine leukemia virus-based retroviral vector pMXs-IRES-Puro or pMXs-IRES-Neo (Cell Biolabs). To generate the retrovirus, Plat-GP cells (Cell Biolabs) were cotransfected with pMXs-IRES-Puro along with an expression vector for VSV-G by using Lipofectamine 2000 (Invitrogen). Two days later, the culture supernatants containing the retroviruses were collected, clarified through 0.45-μm-pore filters, and then used to infect the BHK cells. Stable cells were selected with 4 μg/ml puromycin and/or 300 μg/ml G418 (InvivoGen). All BHK cell lines (wild type and those encoding FcγRIA, FcγRIIA, FcγRIIIA, hACE2, FcγRIA/FCER1G, or FcγRIIIA/FCER1G) were grown in Eagle's minimum essential medium (EMEM) containing 10% FBS and antibiotics with or without puromycin and G418. HEK-293T cells were grown in high-glucose Dulbecco’s modified Eagle’s medium (DMEM) containing 10% fetal bovine serum (FBS), l-glutamine, and antibiotics. Human peripheral blood mononuclear cells (PBMCs) were purchased from Zenbio or Cellular Technology Ltd. PBMCs were cultured in monocyte attachment medium (Promocell) for macrophage development in culture plates coated with ε-poly-l-lysine coating solution (Cosmo Bio). The PBMCs were incubated for 1 to 1.5 h in the incubator without any further manipulation. Attached cells were washed three times with monocyte attachment medium. Monocytes/macrophages were cultured in RPMI containing 10% FBS and antibiotics. All cells were maintained at 37°C and 5% CO_2_.

### Viruses.

A VSV possessing the firefly luciferase gene in place of the VSV-G gene and pseudotyped with SARS-CoV-2 S (VSV-SARS2-S) and a control luciferase-expressing VSV containing only VSV-G were prepared. To generate VSV-SARS2-S, HEK-293T cells were transfected for 24 h with a SARS-CoV-2 S expression vector (SinoBiological) and then were infected with VSV-G at an MOI of >1.0. Supernatant was collected at 24 h postinfection, clarified through 0.45-μm-pore filters, and then used for experiments.

The SARS-CoV-2 isolate NCGM02 was propagated in Vero E6 cells in Opti-MEM I (Invitrogen) containing 0.3% bovine serum albumin (BSA) and 1 μg of l-1-tosylamide-2-phenylethyl chloromethyl ketone treated-trypsin per ml or in Vero 76 cells in Eagle’s minimal essential medium supplemented with 2% fetal calf serum at 37°C. SARS-CoV-2 variants (QHN001, TY7-501, and TY8-612) were propagated in Vero E6/TMPRSS2 cells in VP-SFM (Thermo Fisher Scientific).

All experiments with SARS-CoV-2 were performed in enhanced biosafety level 3 (BSL3) containment laboratories at the University of Tokyo, which are approved for such use by the Ministry of Agriculture, Forestry, and Fisheries, Japan, or in enhanced BSL3 containment laboratories at the University of Wisconsin—Madison, which are approved for such use by the Centers for Disease Control and Prevention and by the U.S. Department of Agriculture.

### Assessment of cell entry.

To examine cell entry mediated by SARS-CoV-2 S, BHK cell lines (wild type and those encoding FcγRIA, FcγRIIA, FcγRIIIA, or hACE2) were seeded into 96-well tissue culture plates. A neutralizing monoclonal antibody against the VSV-G protein (clone I-1) was used to abolish the background infectivity of parental VSV-G virus in the virus stock of VSV-SARS2-S. Cells were then infected with VSV-SARS2-S virus. Twenty-four hours later, cells were lysed and analyzed for firefly luciferase activity by using the Steady-Glo luciferase assay system (Promega) according to the manufacturer’s instructions.

### Evaluation of ADE.

To examine cell entry of SARS-CoV-2 via antibodies from patients, BHK-hACE2, BHK-FcγRIA, BHK-FcγRIIA, BHK-FcγRIIIA, BHK-FcγRIIA/FCER1G, or BHK-FcγRIIIA/FCER1G cells were seeded into 96-well tissue culture plates with or without transfection of a vector encoding hACE2. VSV-SARS2-S was treated with a neutralizing monoclonal antibody against the VSV-G protein (clone I-1) to abolish the background infectivity of parental VSV-G virus. Plasma samples were heat inactivated for 30 min at 56°C and serially diluted (from 1:25 to 1:25,600) before being incubated with VSV-SARS2-S for 1 h at 37°C. The virus-plasma mixture was then added to the indicated cells and incubated at 37°C. Twenty-four hours later, cells were lysed and analyzed for firefly luciferase activity by using the Steady-Glo luciferase assay system (Promega) according to the manufacturer’s instructions. Monocytes-derived macrophages were plated in 24-well plates coated with ε-poly-l-lysine coating solution and were infected and analyzed as described above.

### RT-qPCR.

Total RNA was isolated from the cells by using the RNeasy minikit (Qiagen, Tokyo, Japan). To quantify SARS-CoV-2 N genes, one-step RT-qPCR was performed using the LightCycler 96 system (Roche Diagnostics, Tokyo, Japan) according to the protocol of the National Institute of Infectious Disease, Japan (30). The One Step TB Green PrimeScript RT-PCR kit II (TaKaRa, Tokyo, Japan) was used to quantify *GAPDH*, which was used for normalization. The primers used for *GAPDH* were 5′-TGCACCACCAACTGCTTAGC-3′ (forward) and 5′-ATGGCATGGACTGTGGTCATGAG-3′ (reverse).

### Cytokine assay.

The Bio-Plex Pro human cytokine 27-plex assay (Bio-Rad) was used to quantify the cytokines in the supernatant of the macrophages. Bio-Plex 200 Systems (Bio-Rad) were used according to the manufacturer’s instructions.

### Statistical analysis.

Statistical analysis was performed by using GraphPad Prism 9.1.1. *P* values were considered significant if they were less than 0.05. The statistical analysis method used and the number of biological replicates and technical replicates for each experiment are described in each figure legend. 
