# Supplementary figures and images for: Antibody-Dependent Enhancement of SARS-CoV-2 Infection Is Mediated by the IgG Receptors FcγRIIA and FcγRIIIA but Does Not Contribute to Aberrant Cytokine Production by Macrophages
Source: mBio. 2021 Sep 28;12(5):e01987-21. doi: 10.1128/mBio.01987-21 (PMC8546849; doi:10.1128/mBio.01987-21)

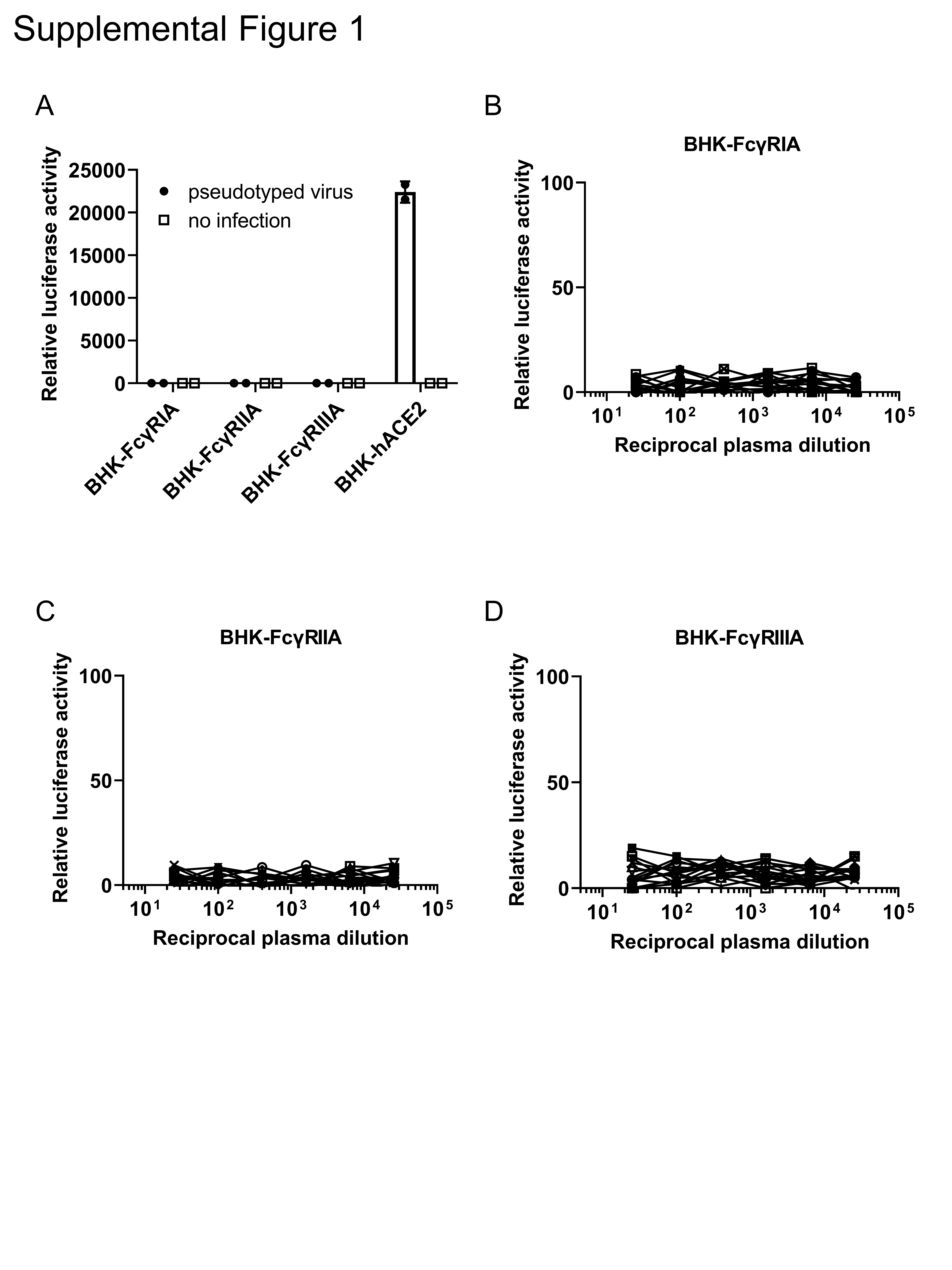

Supplement: FIG S1 [file mbio.01987-21-sf001.tif]

# Supplemental Figure 2A

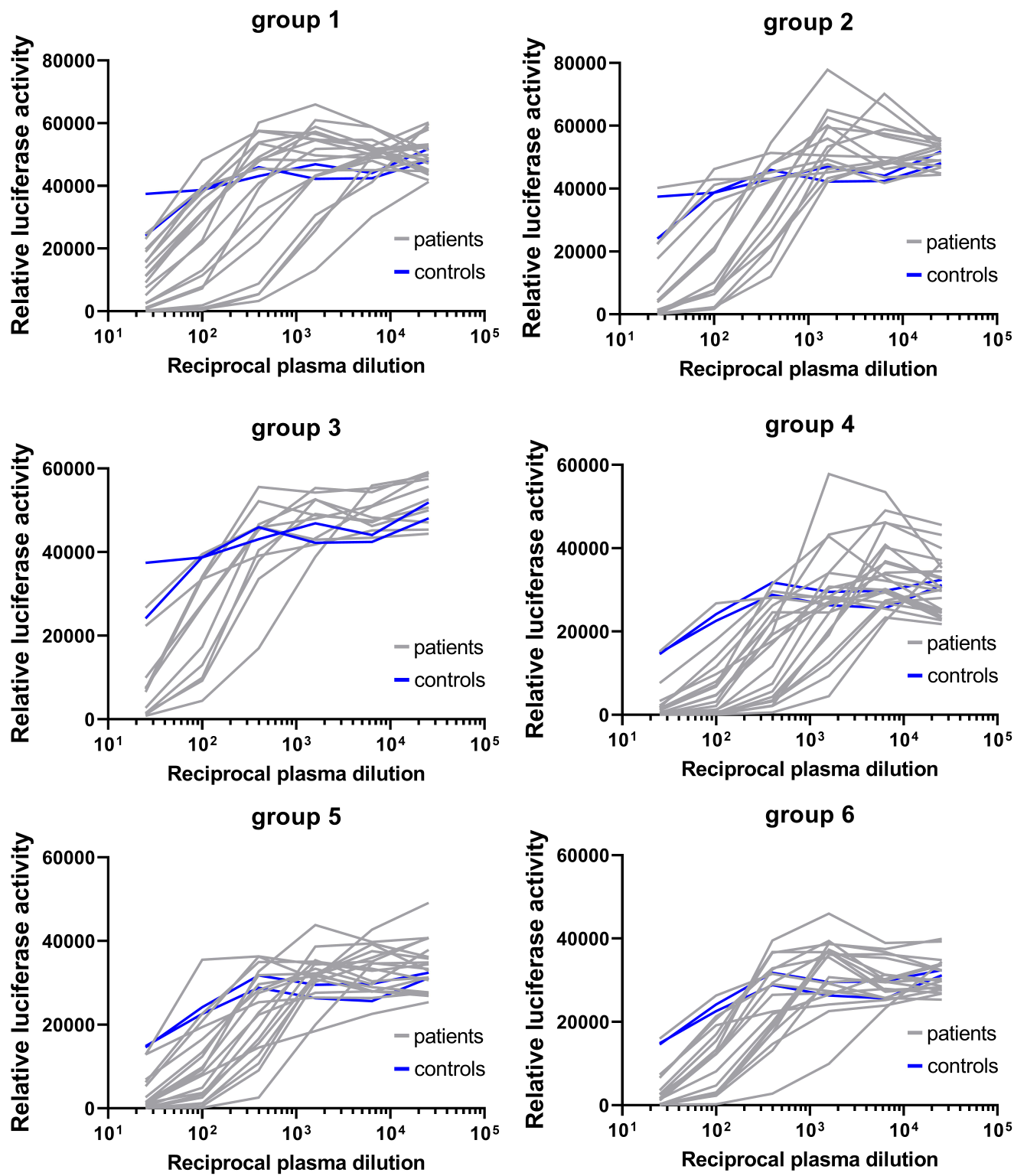

Supplemental Figure 2B

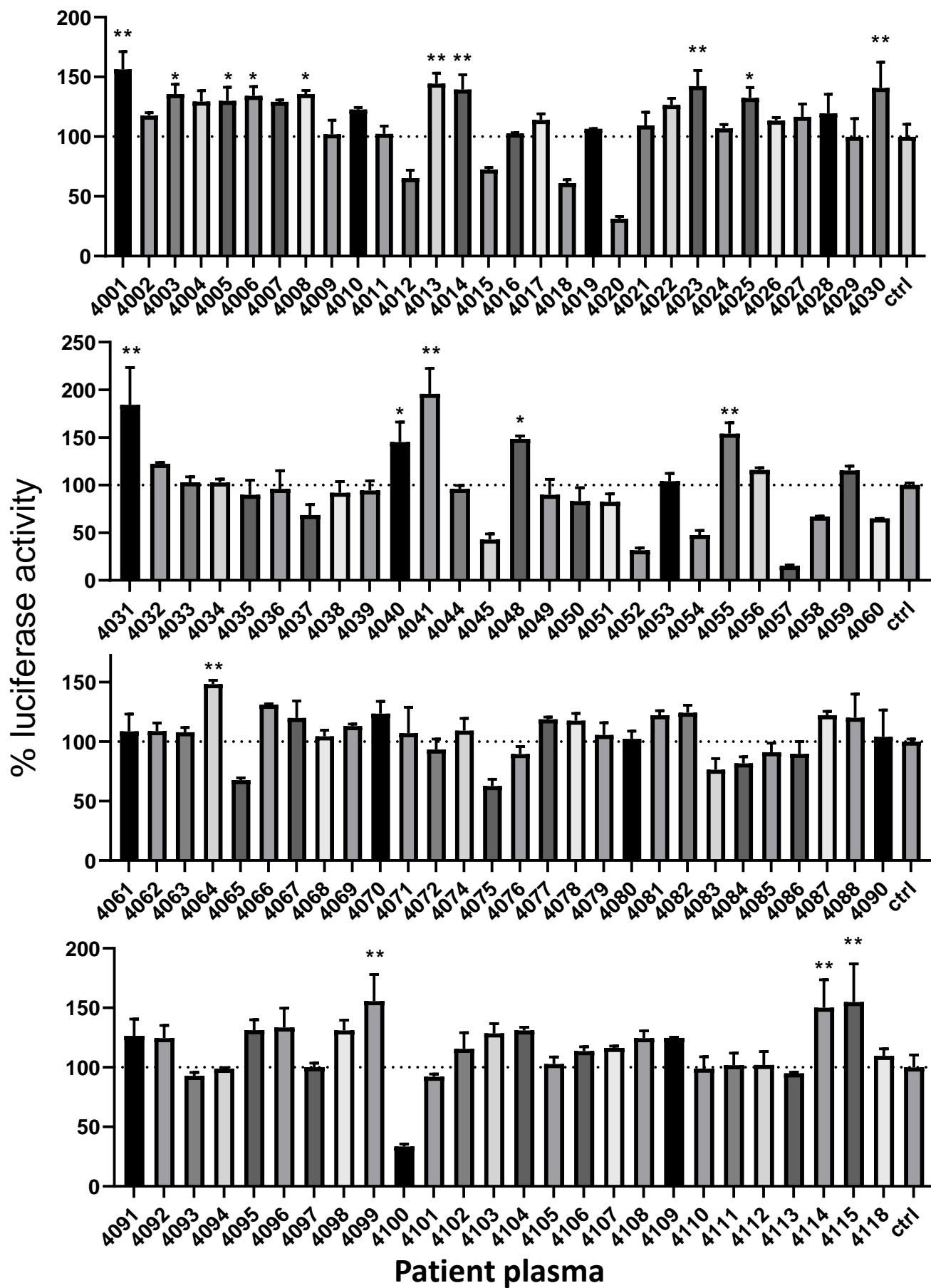

Supplemental Figure 2C

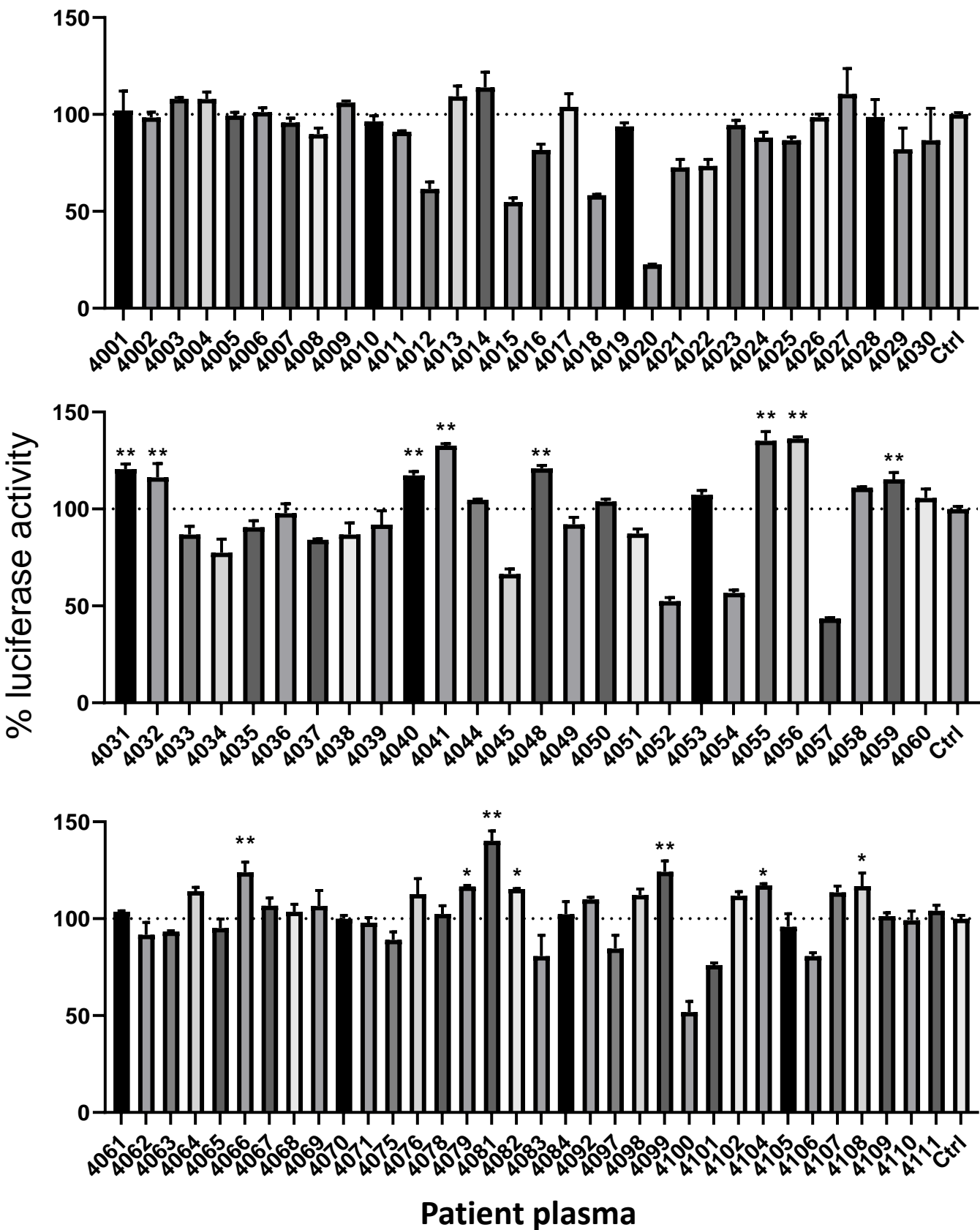

Supplement: FIG S2 [file mbio.01987-21-sf002.pdf]
